# Investigation of Retinal Metabolic Function in Type 1 Diabetic Akita Mice

**DOI:** 10.3389/fcvm.2022.900640

**Published:** 2022-06-02

**Authors:** Esraa Shosha, Luke Qin, Tahira Lemtalsi, Syed A. H. Zaidi, Modesto Rojas, Zhimin Xu, Robert William Caldwell, Ruth B. Caldwell, Abdelrahman Y. Fouda

**Affiliations:** ^1^Vascular Biology Center, Augusta University, Augusta, GA, United States; ^2^Department of Clinical Pharmacy, Faculty of Pharmacy, Cairo University, Giza, Egypt; ^3^University of Arkansas for Medical Sciences, Little Rock, AR, United States; ^4^Culver Vision Discovery Institute, Augusta University, Augusta, GA, United States; ^5^Department of Pharmacology and Toxicology, Augusta University, Augusta, GA, United States

**Keywords:** diabetic retinopathy, Akita mice, type 1 diabetes, retinal metabolic function, Seahorse, glycolysis, mitochondrial respiration

## Abstract

Diabetic retinopathy (DR) is the leading cause of vision loss in working age adults. Understanding the retinal metabolic response to circulating high glucose levels in diabetic patients is critical for development of new therapeutics to treat DR. Measuring retinal metabolic function using the Seahorse analyzer is a promising technique to investigate the effect of hyperglycemia on retinal glycolysis and mitochondrial respiration. Here, we analyzed the retinal metabolic function in young and old diabetic and control mice. We also compared the expression of key glycolytic enzymes between the two groups. The Seahorse XF analyzer was used to measure the metabolic function of retina explants from young and old type 1 diabetic Akita (*Ins2^Akita^*) mice and their control littermates. Rate-limiting glycolytic enzymes were analyzed in retina lysates from the two age groups by Western blotting. Retinas from young adult Akita mice showed a decreased glycolytic response as compared to control littermates. However, this was not observed in the older mice. Western blotting analysis showed decreased expression of the glycolytic enzyme PFKFB3 in the young Akita mice retinas. Measurement of the oxygen consumption rate showed no difference in retinal mitochondrial respiration between Akita and WT littermates under normal glucose conditions *ex vivo* despite mitochondrial fragmentation in the Akita retinas as examined by electron microscopy. However, Akita mice retinas showed decreased mitochondrial respiration under glucose-free conditions. In conclusion, diabetic retinas display a decreased glycolytic response during the early course of diabetes which is accompanied by a reduction in PFKFB3. Diabetic retinas exhibit decreased mitochondrial respiration under glucose deprivation.

## Introduction

Diabetic retinopathy (DR) is the leading cause of blindness in working-aged adults (20–74 years) (1, 2). DR is identified in one third of diabetic patients and it is strongly associated with poor blood glucose control (1, 2). Large clinical trials have consistently shown the benefit of tight blood glucose control in reducing the risk of retinopathy development and progression in patients with diabetes (3). Furthermore, development of DR is directly related to the duration of diabetes, and it is more prevalent in type 1 diabetic patients who develop severe diabetes earlier in their lives versus type 2 diabetics who develop diabetes at a later age (4–7). Interestingly, rapid improvement in systemic glucose control following initiation of effective treatment leads to transient worsening of diabetic retinopathy in patients with long-term uncontrolled diabetes (8, 9). Collectively, retina pathophysiology in DR is directly related to glycemia and likely involves intrinsic adaptation to chronic blood glucose levels.

There is a lack of understanding of the pathophysiology of DR and the retinal metabolic response to circulating high glucose levels that could be a driving factor in the disease progression. Identify abnormalities in retinal metabolism in the early and late stages of DR will aid in the development of new strategies to halt or reverse the retinal injury and prevent blindness.

The heterozygous *Ins2^Akita^* mice have a mutation of the *Insulin 2* gene that causes protein misfolding and pancreatic β-cell degeneration. The Akita mice become hyperglycemic and diabetic at 1 month of age due to reduced insulin levels (10, 11). Therefore, this model has a rapid onset and closely resembles the natural course of human type 1 diabetes. It is useful for studying DR progression and the neuroprotective effects of treatments (12). Another widely used model of type 1 diabetes is streptozotocin (STZ) induced diabetes, where pancreatic β-cells are destroyed by the chemical toxin, STZ (13). DR pathology and retinal functional changes shown by electroretinography (ERG) have been well-documented in both Akita and STZ diabetic mice. Mice with STZ diabetes display decreases in retinal ERG responses (a- and b- waves) as early as 4–8 weeks after diabetes induction (14–16). This contrasts with Akita mice which develop ERG changes at 5–9 months of age (17–20), suggesting a more progressive DR pathology in the STZ mice.

The Seahorse XF Analyzer is a powerful tool to analyze mitochondrial respiration and glycolysis in living cells by measuring oxygen consumption rate (OCR) and extracellular acidification rate (ECAR), respectively. While the company has established a protocol to conduct the technique on pancreatic islets using islet capture microplates, the technique has not been rigorously assessed for other tissue types. Recent elegant studies have adapted the islet capture microplates to measure the metabolic function in retina explants (21–23). In this study, we examined the metabolic function in retina explants from 7 to 14 months old *Ins2^Akita^* mice to determine the metabolic changes in the early and late phases of diabetes. Furthermore, protein expression of key glycolysis enzymes was examined in the *Ins2^Akita^* as well as STZ-induced diabetic mice. Lastly, Seahorse and glycolysis enzyme expression analyses were conducted on an acute model of retinal ischemia-reperfusion (IR) injury that is often used to mimic diabetic retinopathy pathogenesis.

## Materials and Methods

### Mouse Models of Type 1 Diabetes

*In vivo* experiments were performed in accordance with the ARVO Statement for the Use of Animals in Ophthalmic and Vision Research and were approved by the institutional animal care and use committee (Animal Welfare Assurance no. D16-00197).

Akita type 1 diabetic mice (*Ins2^Akita^*) and littermate control C57BL/6J mice were bred in our facility. Animal were sacrificed at different ages for experimental endpoints and littermates from the same cage were compared. Diabetes in our Akita mice was confirmed by blood glucose measurement and genotyping as we previously described (24). Only male Akita mice were used since females exhibited only mild hyperglycemia in our hands (blood glucose **≈** 300 mg/dL vs. an average of 600 mg/dL in males, Supplementary Figure 1A) which is in line with previous reports that suggested a protective effect of estrogen in females (25–27): Male mice also exhibited retinal thinning and ganglion cell loss (Supplementary Figures 1B,C). Before sacrifice, blood glucose levels of Akita mice were measured with an AlphaTRAK 2 glucometer using tail-tip blood. Mice with high blood glucose (>500 mg/dL) were considered diabetic and used in the study along with their littermate controls (blood glucose <300 mg/dL). We noticed that at old age (14–18 months), some Akita mice were moribund or exhibited weight loss and low blood glucose. These mice were euthanized and excluded from the study.

In addition to the Akita model, we used the STZ-induced diabetes and retinal IR-injury models which are explained in detail in the Supplementary Material.

### Seahorse Analysis on Retina Explants

#### Retina Explant Preparation

Mice were anesthetized using ketamine/xylazine and retinas were gently eviscerated and kept in a cell culture dish containing Hank’s balanced salt solution (HBSS). Retina punches of 1 mm diameter were taken from the central area of the retina cups (excluding the optic nerve) under a microscope using 1 mm biopsy punches with plunger (Integra Miltex). Punches were transferred to a Seahorse XF24 islet capture microplate (one punch per well) using a 1 mL pipette with a cut tip to make a wider bore. The temperature of the solutions and the tissues were kept at 37°C during the isolation process using a water bath and heating pad, respectively. Punches were adjusted under the microscope to make the ganglion cell layer facing up. Pre-soaked Seahorse capture screen inserts were placed in each well and secured in place by pressing on two opposite sides using a forceps. We found that a regular pair of forceps is easier to use than the Seahorse capture screen insert tool from Agilent. The islet capture plate with isolated punches was then incubated in a non-CO_2_ incubator for 45–60 min at 37°C before running the assay.

#### Seahorse Run and Injection Protocol

Extracellular acidification rate (ECAR as a measure of glycolysis) and oxygen consumption rate (OCR as a measure of mitochondrial respiration) were assessed on different cohorts of mice using Seahorse XFe24 Analyzer (Agilent, Santa Clara, CA, United States). Retinal punches were prepared on the same day of the assay as described above and placed in Seahorse XF24 islet capture plates. Two wells were left empty to be used as background wells.

Extracellular acidification rate was conducted using a glycolysis stress kit (Agilent, Catalog # 103020-100, Santa Clara, CA, United States). We used the glucose-free Seahorse media (Seahorse XF DMEM Medium, pH 7.4) supplemented with 2 mM glutamine (Agilent). Then, the test was run according to the manufacturer’s instructions with a few modifications in the concentration of injection compounds and ports. We injected vehicle (media only) in port A to exclude false ECAR changes resulting from tissue movements after the first injection as described previously (22). This was followed by glucose (15 mM) in port B, then oligomycin (1 mM) in port C and finally 2-deoxy glucose (75 mM) in port D according to the manufacturer’s protocol.

Oxygen consumption rate was conducted using a Mito Stress Kit (Agilent, Catalog # 103015-100, Santa Clara, CA, United States) according to the manufacturer’s instructions with a few modifications. Seahorse media (Seahorse XF DMEM Medium, pH 7.4) were prepared according to the manufacturer’s instructions and supplemented with 1 mM pyruvate (Agilent) and 5 mM glucose (Sigma-Aldrich, St. Louis, MO, United States). The concentrations of the injection compounds were as follows: Oligomycin (1 μM), FCCP (1 μM) and Rotenone/antimycin A (0.5 μM). In port A, we injected vehicle as described above followed by the injection of oligomycin in port B, FCCP in port C and rotenone/antimycin A in port D. We used an assay protocol of mixing time (2 min) followed by waiting time (2 min) and then measuring for 5 min. That was repeated for 5 cycles for each measurement point. The data were collected and analyzed using the Wave software (Agilent, Santa Clara, CA, United States) and normalized to protein content.

#### Protein Estimation and Normalization

At the end of the ECAR and OCR assays, the Seahorse media was removed and 1x RIPA lysis buffer (Thermo Fisher Scientific, Waltham, MA, United States, cat# 89900) (50 μl per well) was added to each well and then the plate was frozen at −80°C. The next day, the plate was allowed to thaw and retina punches were homogenized by pipetting up and down without removing the mesh. We measured the protein content using Pierce™ BCA Protein Assay Kit (Thermo Fisher Scientific, Waltham, MA, United States, cat# 23225) following the manufacturer’s instructions using 5 μl samples in duplicates. Protein concentration was used for data normalization in the Wave software.

#### Data Analysis and Representation

In each experiment we used 1–2 mice (2–4 retinas) per experimental group, and we isolated the punches from those retinas to achieve the replicates within each run (2–4 punches per retina). The experiment was then repeated on a different day using mice of the same age range to account for biological variability between groups and variations due to the Seahorse reagents and machine. We found slight differences in baseline and responses to injection compounds from one run to another but the trend between experimental groups remained largely the same. We tried to pool different plates together using the Multi-File XF Report Generator (Agilent). We found that pooling different runs together increases the variability within experimental groups and therefore only one representative run is presented and statistically analyzed for each experimental timepoint.

### Retina Tissue Collection and Western Blotting

Mice were deeply anesthetized, and retinas were dissected and snap frozen at −80°C. Retinas were later homogenized in Radioimmunoprecipitation Assay (RIPA) lysis buffer (Millipore, Billerica, MA, United States) containing 1X protease and phosphatase inhibitors (Complete Mini and phosSTOP, respectively; Roche Applied Science, Indianapolis, IN, United States) using a micro tube handheld tissue homogenizer. Western blotting on retina lysates was conducted as previously described (28). Membranes were probed with the primary antibodies followed by HRP-linked secondary antibody. Data were quantified by densitometry using ImageJ and normalized to loading control. The following antibodies were used in the study: PKM2 (Cell Signaling, Danvers, MA, United States, cat# 4053), PDH (Cell Signaling, Danvers, MA, United States, cat# 3205), HK1 (Cell Signaling, Danvers, MA, United States, cat# 2024), HK2 (Cell Signaling, Danvers, MA, United States, cat# 2867), PFKFB3 (Cell Signaling, Danvers, MA, United States, cat# 13123), and anti-rabbit IgG, HRP-linked secondary antibody (Cell Signaling, cat# 7074).

### Electron Microscopy and Quantitative Assessment of Mitochondrial Morphology

Eyeballs from 5-month-old WT and Akita mice were fixed in 4% paraformaldehyde, 2% glutaraldehyde in 0.1 M sodium cacodylate (NaCac) buffer, pH 7.4, postfixed in 2% osmium tetroxide in NaCac, and stained en bloc with 2% uranyl acetate, dehydrated with a graded ethanol series, and embedded in Epon-Araldite resin. Thin sections of the retina were cut with a diamond knife on a Leica EM UC7 ultramicrotome (Leica Microsystems, Inc., Bannockburn, IL, United States), collected on copper grids and stained with uranyl acetate and lead citrate. Tissue was observed in a JEM-1400 Flash transmission electron microscope (JEOL USA, Inc., Peabody, MA, United States) at 120 kV and imaged with an One View Digital Camera Controller (Gatan Inc., Pleasanton, CA, United States). Retinas were analyzed for the morphology of their mitochondria. Multiple images were taken from the photoreceptor inner segment layer using electron microscopy. For each mouse, averaged data points for each parameter (perimeter and circularity) were calculated as follows: Images were imported into Adobe Photoshop to assess each mitochondrion shown in view while excluding mitochondria that were partially cut off from frame. Photoshop’s free form cropping tool was used to select out each mitochondrion. The software’s measurement tool was implemented to obtain measurements of each cropped mitochondria’s perimeter, and circularity. Circularity is a function of area and perimeter [Circularity = (4 × π) × (Area/Perimeter^2^)] which compares a two-dimensional projection of an object to a circle, where a value of 1.0 is a perfect circle and a value of 0 is a straight line.

### Statistical Analysis

Statistical analysis was conducted using GraphPad Prism 9 software. Differences between two groups were determined by student’s *t*-test. *P*-values < 0.05 were considered statistically significant. Graphs were prepared using GraphPad Prism 9 software and data were presented as mean ± standard error (SE).

## Results

### Measuring Glycolytic Function of the Diabetic Retina

Mice heterozygous for the Akita spontaneous mutation (*Ins2^Akita^*) develop hyperglycemia around 4 weeks of age. We have previously characterized the diabetic phenotype in our established colony of *Ins2^Akita^* mice (24). These mice remain hyperglycemic throughout their life span until they became moribund by 16–18 months of age which is evident by severe weight loss, decreased mobility and low blood glucose. Therefore, we performed our experiments at 7 and 14 months of age (middle-aged and old groups, respectively).

**FIGURE 1:**
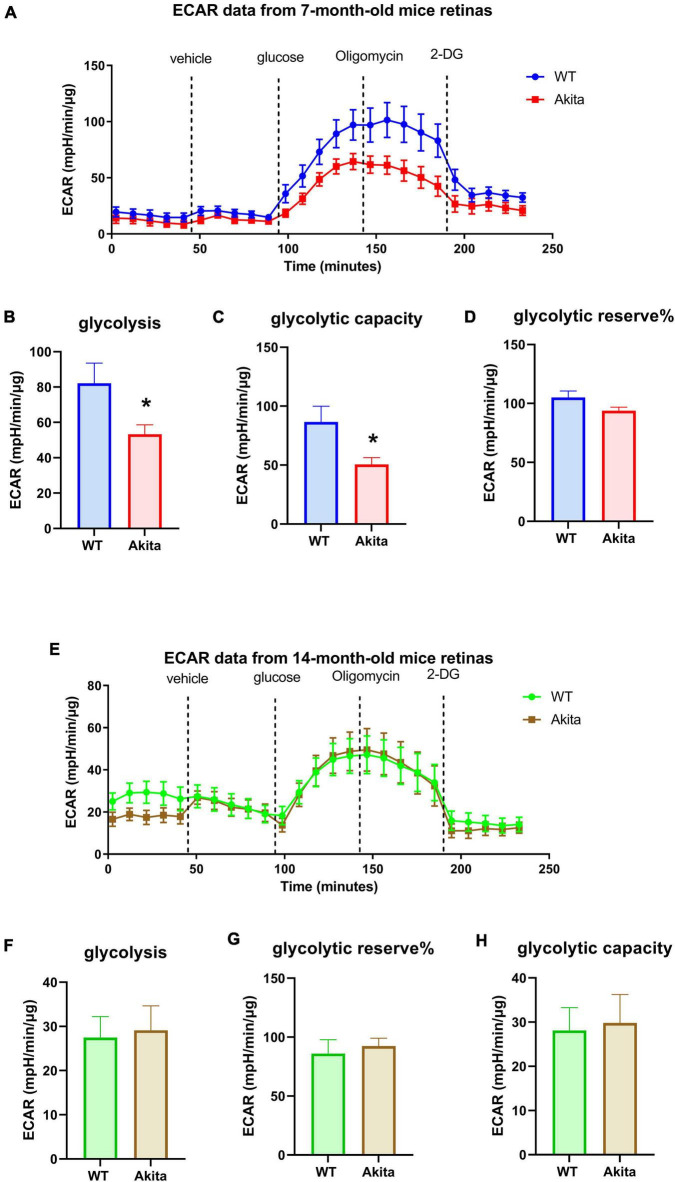
Glycolytic response of the *Ins2^Akita^* diabetic retina. **(A–D)** Seahorse glycolysis stress test conducted on retina explants from 7 months old WT and diabetic Akita mice shows a significant reduction in glycolysis and glycolytic capacity, but not glycolytic reserve in the diabetic retina as indicated by decreases in the extracellular acidification rate (ECAR) compared to the WT controls. *p < 0.05 vs. WT. *N* = 11. **(E–H)** Seahorse glycolysis stress test conducted on diabetic 14-months old retina explants show similar ECAR responses to age-matched WT retinas as presented by similar glycolysis, glycolytic capacity and glycolytic reserve. *N* = 11.

We used the Seahorse XFe24 Analyzer to measure glycolysis (extracellular acidification rate or ECAR) and mitochondrial respiration (oxygen consumption rate or OCR) in retina explants from control and diabetic Akita mice. Measuring ECAR at 7 months of age showed a decrease in glycolysis in response to 15 mM glucose injection in Akita mice as compared to littermate controls (Figures 1A,B). Interestingly, oligomycin, which inhibits mitochondrial ATP production and pushes glycolysis to its maximum, led to a slight increase in control retinas and a slight decrease in Akita diabetic retinas. This resulted in a lower calculated glycolytic capacity and glycolytic reserve in Akita mice as compared to controls but the later did not reach statistical significance (Figures 1C,D). Surprisingly, measuring ECAR at 14 months of age did not show differences in glycolysis, glycolytic capacity or glycolytic reserve between the control and diabetic retinas (Figures 1E–H).

### Expression of Rate-Limiting Glycolysis Enzymes in the Diabetic Retina

**FIGURE 2:**
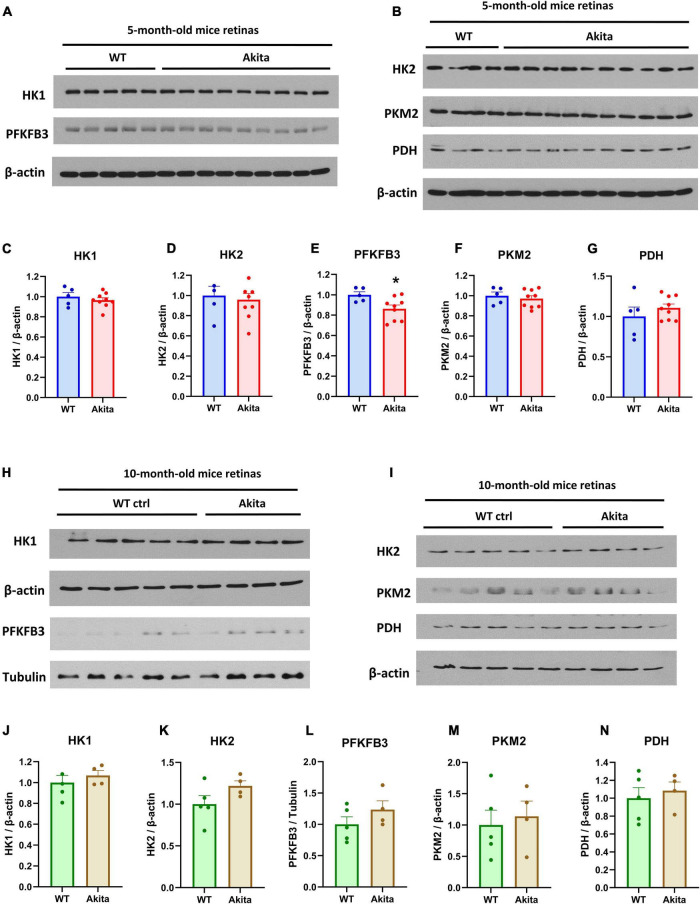
Glycolysis enzymes expression in the *Ins2^Akita^* diabetic retina. **(A–G)** Western blotting analyses and quantification was conducted for the glycolysis enzymes; hexokinase 1 (HK1), 6-phosphofructo-2-kinase/fructose-2,6-biphosphatase 3 (PFKFB3), hexokinase 2 (HK2), pyruvate kinase M2 (PKM2) and Pyruvate dehydrogenase (PDH). The expression of the rate limiting glycolysis enzyme, PFKFB3 shows a significant decrease in retinas from 5-month-old Akita mice while other key glycolytic enzymes remain unchanged. *N* = 5–9. *p < 0.05 vs. WT. Data is presented as fold change of the WT. **(H–N)** Western blotting analyses and quantification of the levels of the glycolytic enzymes mentioned above conducted on 10-month-old Akita mice retinas show no change in key glycolytic enzymes. *N* = 4–5. Data is presented as fold change of the WT.

We next examined retinal protein levels of key enzymes that mediate rate limiting steps in the glycolysis pathway using Western blotting analyses. The glycolysis enzymes, hexokinase 1 (HK1), hexokinase 2 (HK2), pyruvate kinase M2 (PKM2), and pyruvate dehydrogenase (PDH) did not change between the control and Akita diabetic retinas at 5 and 10 months of age (Figures 2A–D,F–K,M,N). Interestingly, the rate limiting glycolysis enzyme 6-phosphofructo-2-kinase (PFKFB3) was significantly decreased in the diabetic Akita retinas as compared to controls at 5 months of age, but there was no statistically significant increase at 10 months of age (Figures 2E,L). To gain insight on the changes in these glycolysis enzymes in another mouse model of type 1 diabetes, we performed Western blotting on retinas from streptozotocin-induced diabetic mice. There was no change in the glycolysis enzymes examined after 8 weeks of diabetes (Supplementary Figures 2A–K,N). However, there was a significant decrease in PKM2 after 17 weeks of diabetes, with PFKFB3 showing a trend toward reduction as well (Supplementary Figures 2L,M).

### Glycolytic Function and Glycolysis Enzymes Expression After Acute Retinal Ischemic Injury

We next examined retinal glycolysis in mice subjected to acute retinal ischemia-reperfusion injury (IR) via increasing the intraocular pressure. The retinal IR-injury is a widely used accelerated model to mimic the ischemic phase of DR. Similar to our findings in the diabetic Akita mice, Seahorse analysis showed a decrease in glycolysis at 24 h after IR as compared to the sham retina, yet the decrease did not reach statistical significance (Supplementary Figures 3A–D). Western blotting analyses of glycolysis enzymes did not show differences between the sham and IR retinas at 6 or 24 h after injury (Supplementary Figure 4).

### Mitochondrial Respiration in the Diabetic Retina

**FIGURE 3:**
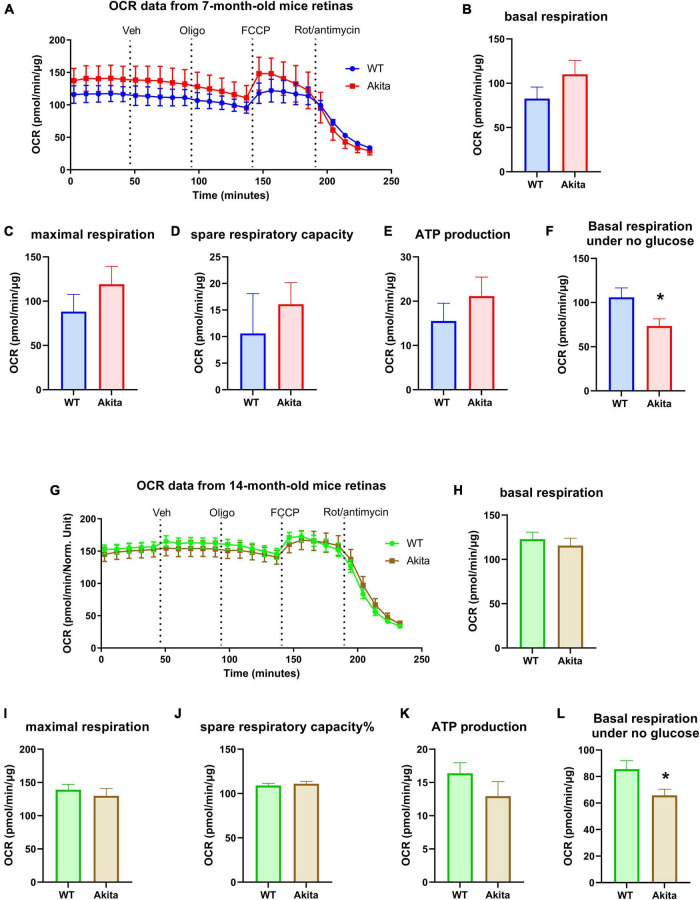
Mitochondrial respiration of the *Ins2^Akita^* diabetic retina. **(A–E)** Seahorse mitochondria stress test conducted by measuring the oxygen consumption rate (OCR) in retinal explants from 7-month-old mice shows no changes in basal respiration, maximal respiration, spare respiratory capacity and ATP production compared to control. *N* = 4. **(G–K)** Seahorse mitochondria stress test conducted by measuring the oxygen consumption rate (OCR) in retinal explants from 14-month-old mice shows no changes in basal respiration, maximal respiration, spare respiratory capacity and ATP production compared to control. *N* = 10–12. **(F,L)** Basal respiration calculated from the glycolysis assay analyses under no glucose conditions shows a reduction in basal OCR in the diabetic retinas from both 7- and 14-month-old Akita mice. *p < 0.05 vs. WT. *N* = 11.

We then measured the mitochondrial respiration in the diabetic retina using the standard Seahorse protocol in 5 mM glucose media. Oxygen consumption rate (OCR) measurement on retinas from 7 months old mice showed no difference in mitochondrial respiration between Akita and WT littermates with the Akita showing a slightly higher trend that was not statistically significant (Figures 3A–E). To our surprise, measuring OCR at 14 months of age still did not show any difference between the two experimental groups in any of the OCR parameters (Figures 3G–K). Taking advantage of the fact that the Seahorse simultaneously measures both ECAR and OCR, we examined the OCR from the glycolysis assay experiments where the retinas were incubated in glucose-free conditions. Interestingly, the Akita retinas showed a significant reduction in basal respiration in both 7 and 14-month old groups (Figures 3F,L). This highlights a differential response of the diabetic retina mitochondrial function under glucose versus glucose-free conditions.

### Electron Microscopic Examination of Mitochondria in the Diabetic Retina

**FIGURE 4:**
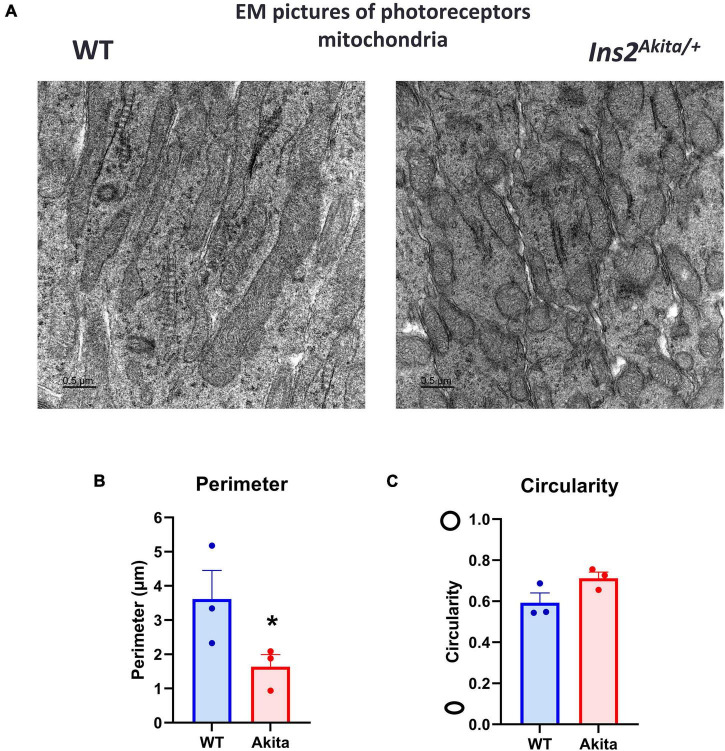
Electron microscopic examination of retinal mitochondrial morphology in diabetes. **(A–C)** Electron microscopic examination of mitochondria in the photoreceptor inner segment layer shows a fragmented morphology in 5-month-old *Ins2^Akita^* diabetic retina as evident by decrease in mitochondria perimeter and increase in circularity yet the later did not reach statistical significance. *N* = 3, *p < 0.05 vs. WT.

Mitochondrial dysfunction has been reported in diabetic Akita mice retinas (29). Since we were not able to detect differences in mitochondrial OCR, we used electron microscopy to examine mitochondrial morphology in the Akita mice retinas. Alterations in photoreceptor function have been shown to contribute to DR development and therefore we examined the mitochondria in these cells (30). Five-month-old Akita mice retinas displayed a fragmented mitochondrial morphology as evident by increased mitochondrial circularity and decreased perimeter (Figures 4A–C). These results suggest that while there were changes in mitochondria structure in the diabetic Akita retinas, Seahorse analysis only detected change in mitochondrial function as measured by OCR under glucose-free conditions.

### Retinal Mitochondrial Respiration After Acute Retinal Ischemic Injury

We then measured retinal mitochondrial function in retinas isolated from mice subjected to IR. Mitochondrial respiration as measured by OCR showed a trend toward reduction after IR as compared to shams. However, none of the calculated parameters were statistically significant (Supplementary Figures 5A–E). Basal respiration calculated under glucose-free conditions from the glycolysis assay showed a trend toward reduction in OCR, but this alteration was not statistically significant (Supplementary Figure 5F).

## Discussion

This study reports for the first time the metabolic response of the diabetic retina from middle aged and old mice as determined by Seahorse analyses of glycolysis and mitochondrial respiration. Our results show a decreased glycolytic response in the diabetic retinas from middle aged Akita mice; however, this difference is no longer present at older age. In line with the decreased glycolysis in middle aged Akita retinas, the rate limiting glycolysis enzyme, PFKFB3, was significantly decreased in the diabetic Akita retinas as compared to controls at 5 months of age but was not different at 10 months of age. The PFKFB3 enzyme synthesizes fructose 2,6-bisphosphate which is the most potent allosteric activator of 6-phosphofructo-1-kinase (PFK-1), thus leading to stimulation of glycolysis (31). Studies from our group and others have shown that PFKFB3 is heavily implicated in retinal angiogenesis (32–35). The current study shows a possible, previously unrecognized depression of PFKFB3 expression in the diabetic retina. Further studies are needed to address the specific role of PFKFB3 in DR.

Our results are in line with a previous report showing accumulation of glucose and decreased lactate production in the diabetic rat retina as measured by NMR spectroscopy, which the authors interpreted as a decrease in glycolytic flux (36). Similarly, metabolite analysis on excised retinas from control and diabetic rats that were exposed to euglycemic and hyperglycemic conditions (5 and 20 mM glucose, respectively) showed decreased glycolytic flux, with no change in the tricarboxylic acid cycle flux in the diabetic retinas under both glycemic conditions (37). One study conducted on vitrectomy samples from patients with or without DR showed that while glucose levels were similar, DR vitreous displayed a decrease in downstream glycolytic metabolites, glyceraldehyde 3-phosphate, 2/3-phosphoglycerate, and the lactate to pyruvate ratio (38).

We compared the changes in retinal glycolysis enzymes in the Akita to STZ-induced diabetes which is also a model of type 1 diabetes. Unlike the Akita, the STZ diabetic retinas showed a decrease in PKM2, another glycolysis rate-controlling enzyme, after 17 weeks but not 8 weeks of diabetes, with PFKFB3 showing a trend toward reduction as well. A recent study reported a decrease in retinal PKM2 expression in 10-week-old type 2 diabetic obese *db/db* mice with no change in the other isoform, PKM1. Interestingly, the same study could not detect a change in PKM1 or PKM2 in type 1 diabetic mouse retinas from STZ diabetic mice that were diabetic for 6 weeks but PDH was increased in these mice (39).

Both the Akita and STZ mice in our colony are on a C57BL/6J genetic background, thus eliminating any possible effect of strain differences on the experimental outcomes. The time points selected in this study (8 and 17 weeks of diabetes for STZ-induced mice and, 5 and 10 months of age for the Akita mice) were based on the reported early changes in retinal ERG responses (a- and b- waves) in the STZ mice (4–8 weeks of diabetes) (14–16), as compared to Akita mice which develop ERG changes at 5–9 months of age (17–20). The reason for the differences in glycolytic enzymes expression between Akita and STZ mice is unclear but could be attributed to differences in the pathophysiology of diabetes development in the two models or to STZ untoward effects. Likewise, a study comparing retinal gene expression between the Akita and STZ models found substantial differences in the gene expression after 3 months of diabetes despite similar body weight, blood glucose and glycosylated hemoglobin (HbA1c) levels between the two models (40).

It remains unclear whether the decreased glycolysis in the diabetic retina is involved in the early pathogenesis of DR. One possible explanation for this decreased glycolysis is glucose shunting into the pentose phosphate pathway (PPP). Activation of the PPP has been reported in vitreous and plasma samples from DR patients (38, 41). Diabetic rat retinas showed similar activation of the PPP when exposed to high glucose (42). The PPP plays an important role in diabetes by providing reducing equivalents to counteract oxidative stress, yet it does not provide ATP to meet the cell energy demand (43). In fact, PFKFB3 inhibition has been shown to shunt glucose from glycolysis to the PPP to enhance the cell antioxidant system (44–47). Hence, our observed decrease in PFKFB3 expression in the diabetic retinas could be a mechanism by which the retina adapts to the increased oxidative stress under diabetic conditions.

Mitochondrial dysfunction is well documented in models of DR and has been recently shown to occur in Akita mice at early stages of diabetes (29, 48). Here we report that Akita mice photoreceptors display mitochondrial fragmented morphology as shown by electron microscopy. This is in line with a recent publication documenting mitochondrial fragmentation in STZ-induced diabetic rat retinas (49). However, we could not detect significant changes in OCR in middle aged or old Akita mice using the standard Seahorse Mito Stress test protocol with media containing 5 mM glucose. This could be explained by the fact that retina cells, especially the photoreceptors, rely mainly on glycolysis (50, 51). Another explanation would be the fact that the Seahorse analysis of retinal metabolic function is only measured under light and not dark conditions. A recent study has shown diurnal mitochondrial dynamics in response to light and darkness to cope with higher energy demands in the later (52). It is possible that mitochondrial respiration in the diabetic retina is impaired in the dark when the metabolic demand is higher. Interestingly, basal OCR measurement of the diabetic retina explant under glucose-free conditions showed a significant reduction in mitochondrial function. This shows that glucose deprivation induced metabolic stress by inhibiting glycolysis which unmasked mitochondrial dysfunction in the diabetic retinas. The detailed mechanisms of this differential response of diabetic retina mitochondrial function under glucose or glucose-deprived conditions are yet to be elucidated. Of note, the glucose-free Seahorse media contains amino acids that can serve as a fuel for mitochondrial oxidative metabolism in absence of glucose (53, 54).

Despite being limited by the lack of dark-adapted measurements, the Seahorse explant analysis is a powerful technique that has been recently introduced in the retina field. Two recent studies using oligomycin in a Seahorse analysis of retina explants showed a robust reduction in OCR (about 50% reduction) (22, 23). Another study showed no effect of oligomycin on ECAR and the authors suggested that it is because glycolysis is the primary metabolic mechanism in retina (21). In our hands, oligomycin only elicited a modest reduction in OCR and a small increase in ECAR. The oligomycin response observed in our experiments was similar to that reported in a recent paper that used optic nerve explants (55). Furthermore, we saw a small effect of FCCP on retina explants which is in line with previous reports suggesting limited retinal mitochondria reserve capacity compared to other tissues (22, 23, 56).

Retinal IR injury is widely used to model retinal ischemia in DR (57, 58). Since this model leads to accelerated neurovascular degeneration within few days, we measured the retina metabolic function at 24 h after IR before a drastic tissue loss happens. The IR retinas showed a slight decrease in both ECAR and OCR while Western blotting analysis at 24 h after IR did not show change in expression of glycolysis enzymes. It is possible that different time points after IR would show more drastic changes.

Collectively, our study employed the Seahorse retina explant assay to report decreased glycolysis in diabetic Akita mice. Decreased PFKFB3 expression could be the underlying cause. On the other hand, OCR measurement failed to show decreased mitochondrial function despite evident mitochondrial fragmentation in electron microscopic images. Future studies are needed to further examine the decrease in glycolysis and role of PFKFB3 in DR.

## Data Availability Statement

The original contributions presented in the study are included in the article/Supplementary Material. Further inquiries can be directed to the corresponding author/s.

## Ethics Statement

The animal study was reviewed and approved by the Augusta University Animal Use Committee.

## Author Contributions

ES and AF designed and performed the experiments and drafted the manuscript. LQ performed the data analysis. TL, SZ, and MR contributed to the experiments. ZX contributed to the induction of diabetes and colony maintenance. RWC provided guidance on experimental design. RBC and AF conceived and supervised the project, provided critical feedback, and revised the final manuscript. All authors contributed to the article and approved the submitted version.

## Conflict of Interest

The authors declare that the research was conducted in the absence of any commercial or financial relationships that could be construed as a potential conflict of interest.

## Publisher’s Note

All claims expressed in this article are solely those of the authors and do not necessarily represent those of their affiliated organizations, or those of the publisher, the editors and the reviewers. Any product that may be evaluated in this article, or claim that may be made by its manufacturer, is not guaranteed or endorsed by the publisher.
