# Supplementary material for: Investigation of Retinal Metabolic Function in Type 1 Diabetic Akita Mice
Source: Front Cardiovasc Med. 2022 Jun 2;9:900640. doi: 10.3389/fcvm.2022.900640 (PMC9201036; doi:10.3389/fcvm.2022.900640)
Supplement: Supplementary file 1 [file Data_Sheet_1.pdf]

Supplementary material for article: 'Investigation of retinal metabolic function in type 1 diabetic Akita mice'

SUPPLEMENTARY MATERIALS AND METHODS

1. STZ type 1 diabetes mouse model:

We induced type 1 diabetes in C57BL/6J (wild type, WT) mice from our in-house colony. Seven to eight-week-old male mice were rendered diabetic by intraperitoneal injection (IP) of streptozotocin (STZ, 60 mg/kg) (Sigma-Aldrich, St. Louis, MO, USA; Cat. # S0130) on five successive days. STZ was dissolved in sodium citrate buffer (0.1 M, pH 4.5). Mice were screened for diabetes beginning five days after the first dose of STZ by assessing urine glucose levels with urine testing strips. Mice with fasting urine glucose levels higher than 500 mg/dL were considered to be diabetic. Studies were performed after 8 and 17 weeks of diabetes (4 and 6 months of age respectively).

Diabetes was confirmed by measuring blood glucose levels using an AlphaTRAK 2 glucometer. Only mice with high blood glucose (> 500 mg/dL) were included.

2. Mouse model of retinal ischemia-reperfusion (IR) injury.

Wild-type (WT) C57BL6J male mice (10-12 weeks old) were used for induction of retinal ischemia-reperfusion (IR) injury. Mice were anesthetized using ketamine/xylazine mixture. Then a needle connected to a raised saline bag was inserted into the anterior chamber of the right eye to raise the pressure and induce ischemia for 40 minutes followed by needle removal to allow for reperfusion as described previously.(Fouda *et al.* Cell Death and Disease 2018) The left eye served as sham control. Mice were sacrificed for experimental analysis at 24 hours after IR injury.

SUPPLEMENTARY FIGURES

Supplementary figure 1:

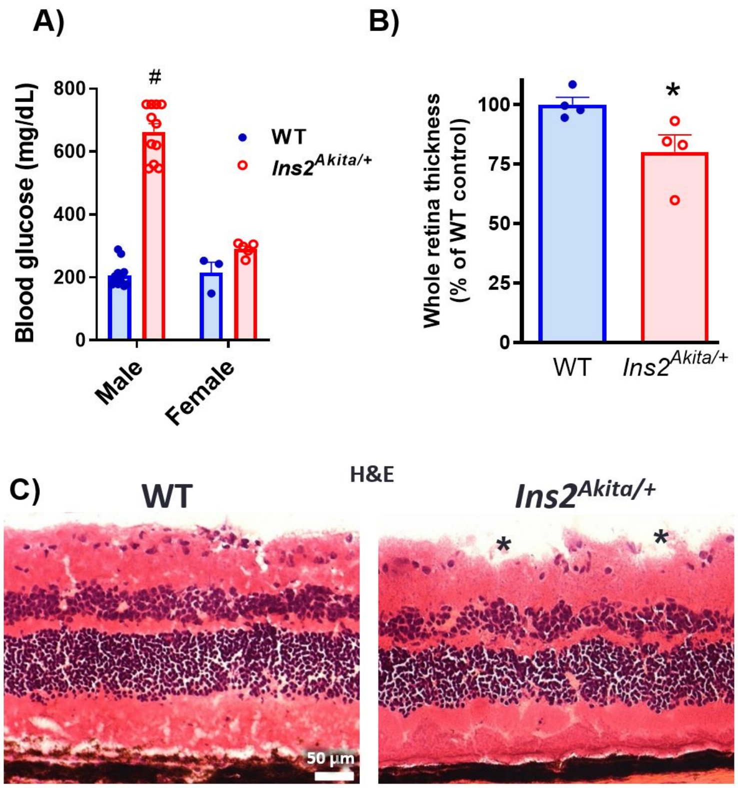

Fig S1: Characterization of the type 1 diabetic *Ins2*$^{Akita/+}$ mice.

(A) Blood glucose measurement showed a significant threefold increase in the 5-month-old male *Ins2*$^{Akita/+}$ diabetic mice as compared to littermate controls. Female *Ins2*$^{Akita/+}$ mice only showed a slight but not significant elevation in blood glucose. N=3-12. $^{\#}$p<0.01 vs WT.

(B, C) Retina sections from male 6-month-old *Ins2*$^{Akita/+}$ mice stained with H&E showed a pathological phenotype with an apparent decrease in ganglion cell number (denoted by asterisk) together with reduced retina thickness. N=4. *p<0.05 vs WT.

Supplementary figure 2:

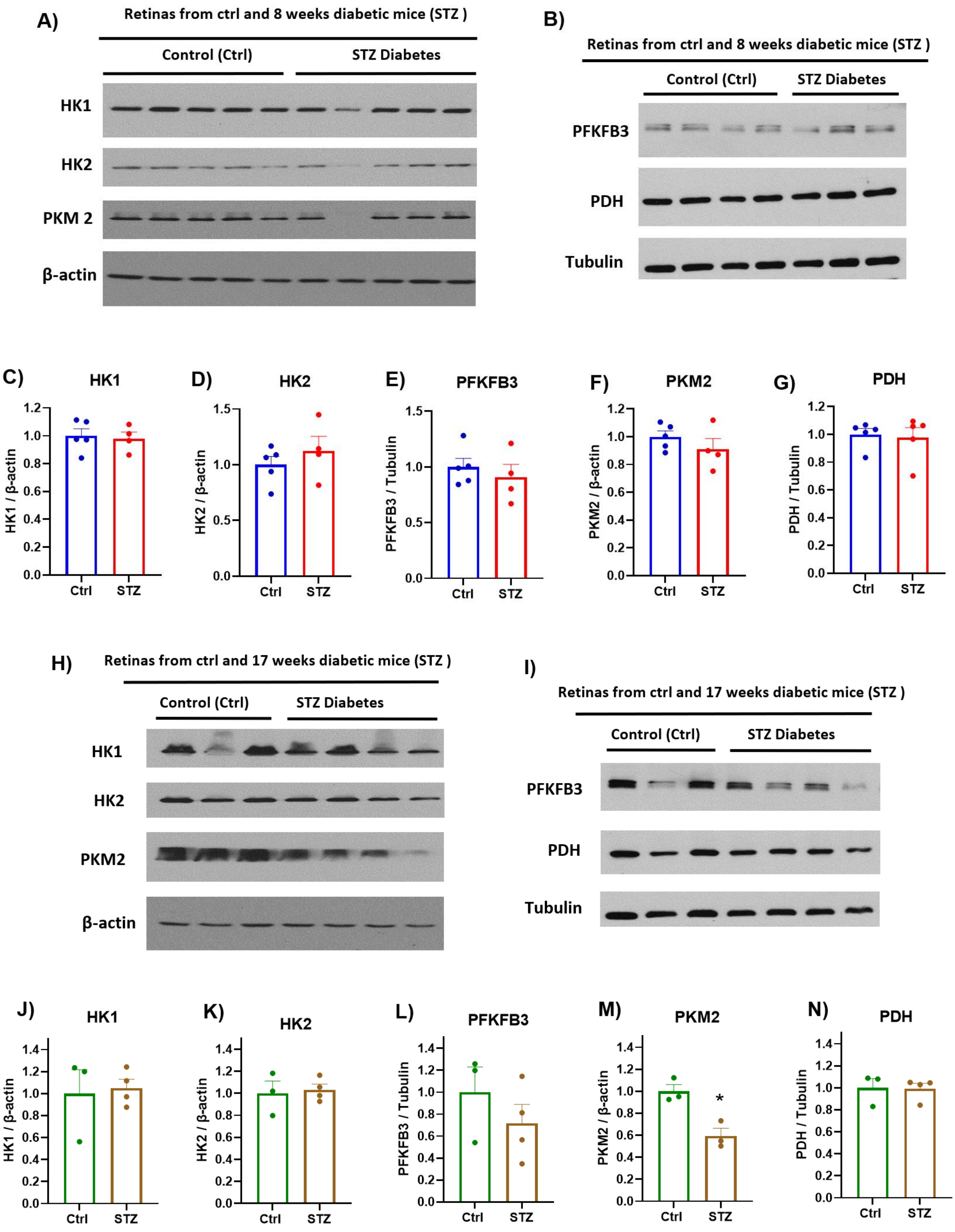

Fig S2: Glycolysis enzymes expression in the STZ-induced diabetic retinas.

A-G) Western blotting on mice retinas collected 8 weeks after STZ-induced diabetes showed no change in glycolysis enzymes expression. N=4-5.

H-N) Western blotting of glycolysis enzymes showed a significant decrease in PKM2 levels in retinas collected 17-weeks after STZ-induced diabetes with only a trend towards reduction in PFKFB3 levels. N=3-4, *$p<0.05$ vs WT.

Supplementary figure 3:

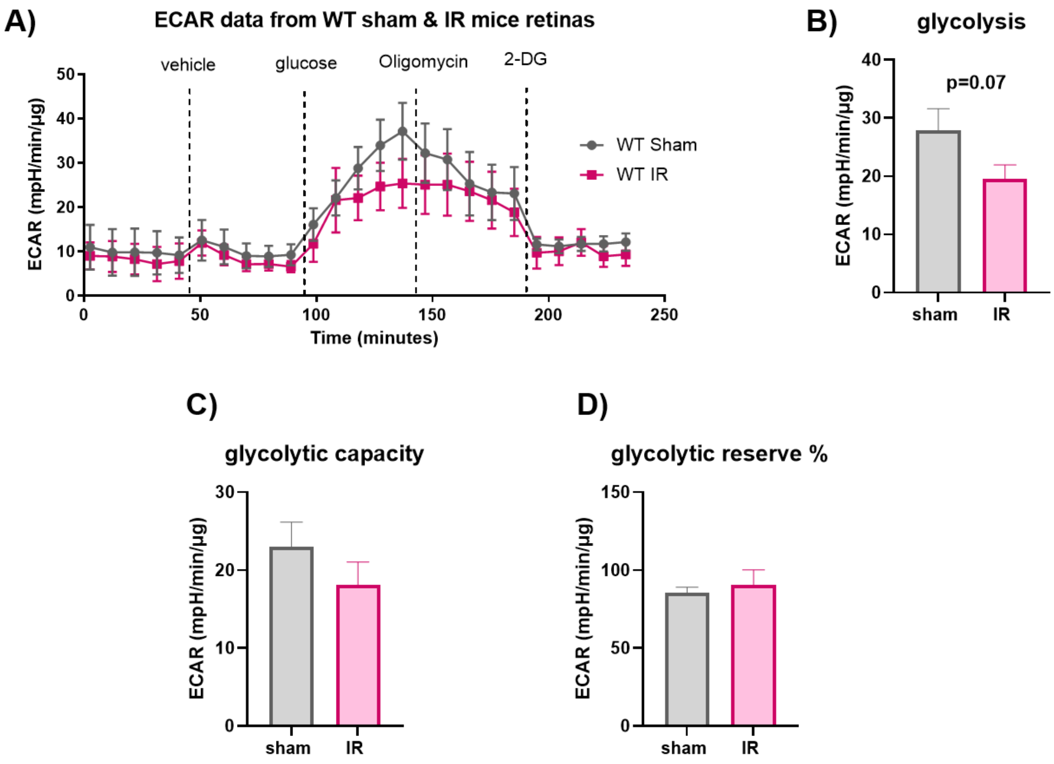

Fig S3: Glycolytic response of retinas subjected to ischemia-reperfusion injury.

A-D) Seahorse glycolysis stress test conducted on retinas isolated 24 hours after sham or IR injury showed a trend towards a reduction in glycolysis ECAR in the IR retinas that was not significantly different from the sham retinas. N=6-8.

Supplementary figure 4:

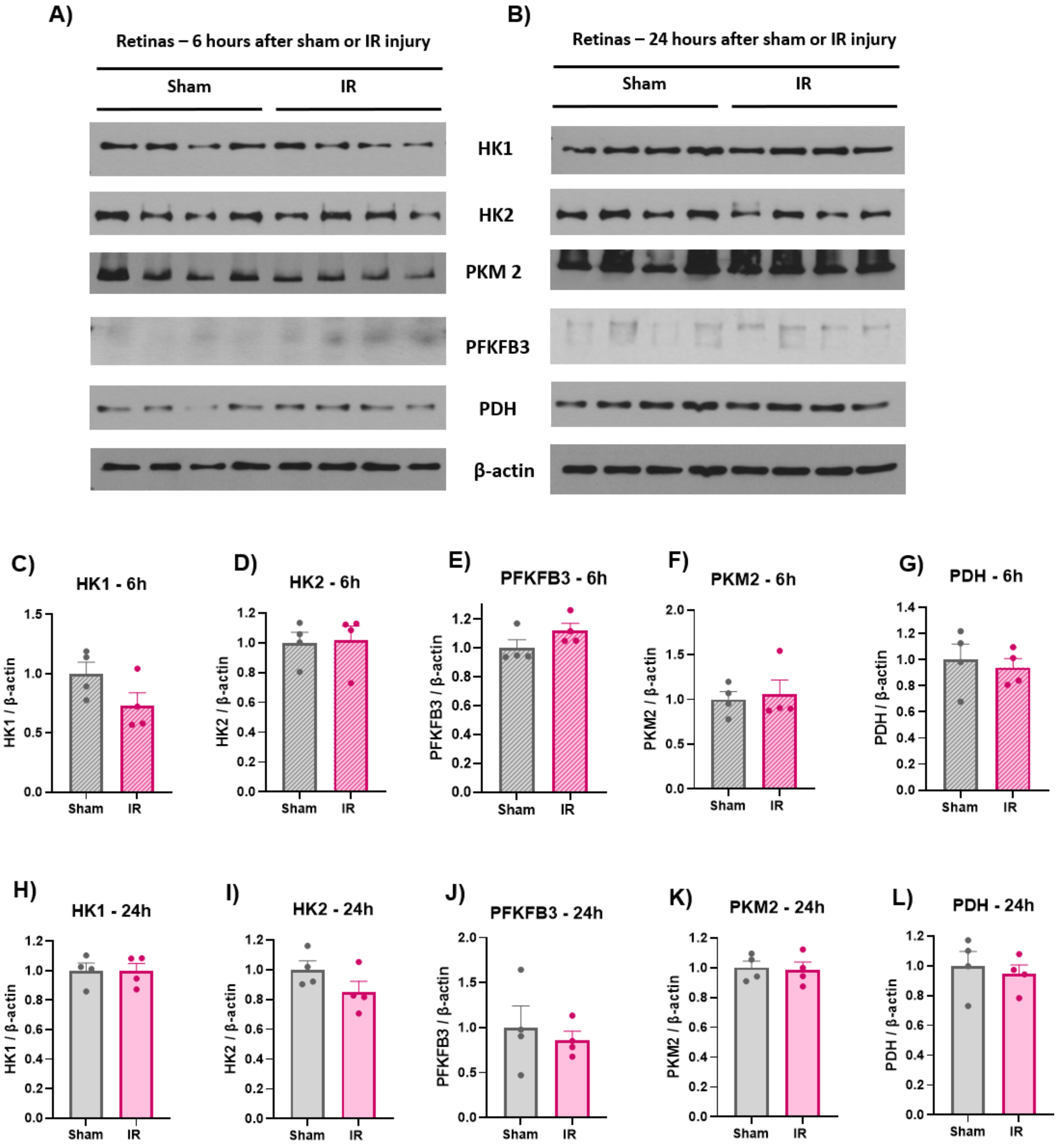

Fig S4: Glycolysis enzymes expression in the retinal ischemia-reperfusion injury model.

(A, C-G) Western blotting analyses of retinal tissues at 6 hours after IR-injury did not show change in glycolysis enzymes expression as compared to sham retinas. N=4.

(B, H-L) Western blotting analyses of retinal tissues at 24 hours after IR-injury did not show any change in glycolysis enzymes expression as compared to sham retinas. N=4.

Supplementary figure 5:

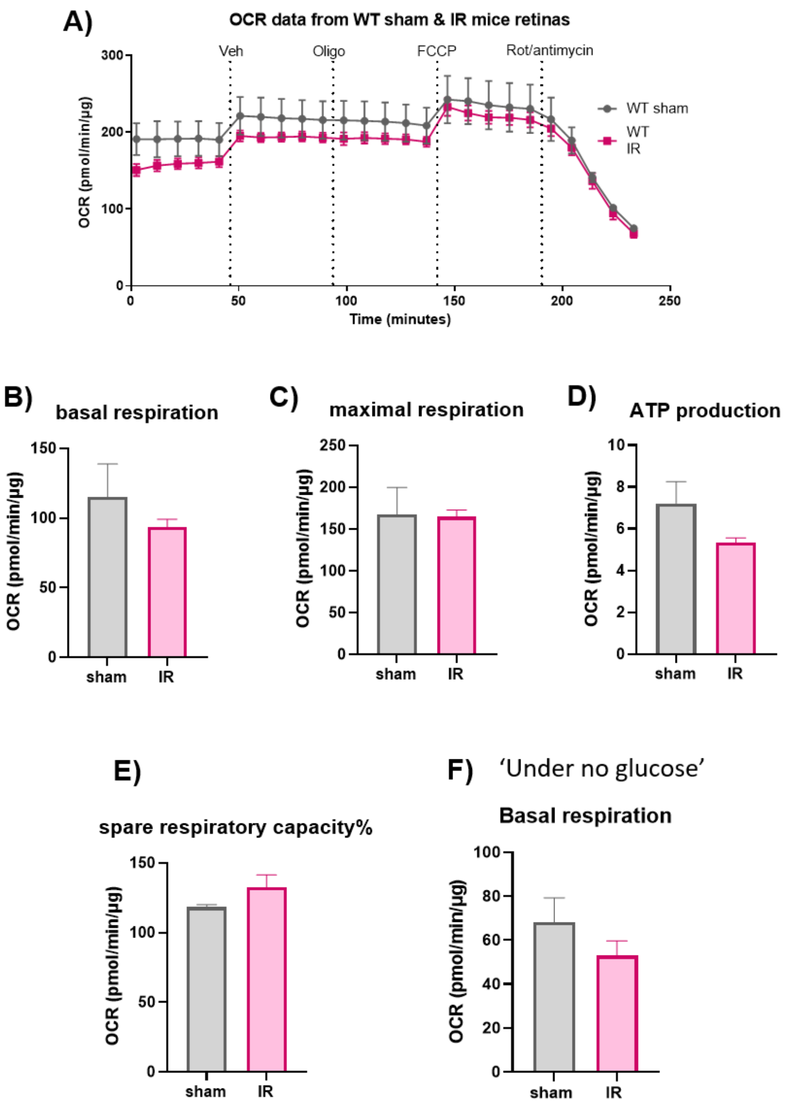

Fig S5: mitochondrial respiration in the retinal ischemia-reperfusion injury model.

(A-F) Seahorse mitochondria stress test conducted on retinas collected after 24 hours of injury showed no change in various parameters of oxygen consumption rate between the sham and IR groups. N=3-4.
